# Correction of a Traffic-Defective Missense ABCB11 Variant Responsible for Progressive Familial Intrahepatic Cholestasis Type 2 †

**DOI:** 10.3390/ijms26115232

**Published:** 2025-05-29

**Authors:** Martine Lapalus, Elodie Mareux, Rachida Amzal, Emmanuelle Drège, Yosra Riahi, Sylvain Petit, Manon Banet, Thomas Falguières, Isabelle Callebaut, Bruno Figadère, Delphine Joseph, Emmanuel Gonzales, Emmanuel Jacquemin

**Affiliations:** 1 Université Paris-Saclay, Inserm, Physiopathogénèse et Traitement des Maladies du Foie, FHU Hepatinov, 91400 Orsay, France; 2 Université Paris-Saclay, CNRS, BioCIS, 91400 Orsay, France; 3 Sorbonne Université, Muséum National d’Histoire Naturelle, CNRS, Institut de Minéralogie, de Physique des Matériaux et de Cosmochimie (IMPMC), 75005 Paris, France; 4 Assistance Publique-Hôpitaux de Paris, Pediatric Hepatology and Pediatric Liver Transplant Department, Reference Center for Rare Pediatric Liver Diseases, FILFOIE, ERN Rare-Liver, Faculté de Médecine Paris-Saclay, CHU Bicêtre, 94270 Le Kremlin-Bicêtre, France

**Keywords:** BSEP, ABC transporter, missense variation, 4-phenylbutyrate, pharmacological correctors

## Abstract

Progressive familial intrahepatic cholestasis type 2 (PFIC2) is a severe hepatocellular cholestasis due to biallelic variations in the *ABCB11* (ATP-binding cassette B11) gene encoding the canalicular bile salt export pump (BSEP). Some missense variants identified in patients with PFIC2 do not traffic properly to the canalicular membrane. However, 4-phenybutyrate (4-PB) has been shown in vitro to partially correct the mis-trafficking of selected variants, resulting in an improvement of the medical conditions of corresponding PFIC2 patients. Herein, we report the ability of 4-PB analogous or homologous drugs and of non-4-PB related chemical correctors to rescue the canalicular expression and the activity of the folding-defective Abcb11^R1128C^ variant. New compounds, either identified by screening a chemical library or designed by structural homology with 4-PB (or its metabolites) and synthesized, were evaluated in vitro for their ability to (i) correct the canalicular localization of Abcb11^R1128C^ after transfection in hepatocellular polarized cell lines; (ii) restore the ^3^H-taurocholate transport of the Abcb11^R1128C^ protein in Madin–Darby canine kidney (MDCK) cells stably co-expressing Abcb11 and the sodium taurocholate co-transporting polypeptide (Ntcp/*Slc10A1*). Glycerol phenylbutyrate (GPB), phenylacetate (PA, the active metabolite of 4-PB), 3-hydroxy-2-methyl-4-phenylbutyrate (HMPB, a 4-PB metabolite analog chemically synthesized in our laboratory) and 4-oxo-1,2,3,4-tetrahydro-naphthalene-carboxylate (OTNC, from the chemical library screening) significantly increased the proportion of canalicular Abcb11^R1128C^ protein. GPB, PA, ursodeoxycholic acid (UDCA), alone or in combination with 4-PB, suberoylanilide hydroxamic acid (SAHA), C18, VX-445, and/or VX-661, significantly corrected both the traffic and the activity of Abcb11^R1128C^. Such correctors could represent new pharmacological insights for improving the condition of patients with ABCB11 deficiency due to missense variations affecting the transporter’s traffic.

## 1. Introduction

Progressive Familial Intrahepatic Cholestasis type 2 (PFIC2), the most severe phenotype associated with ABCB11/BSEP deficiency, is a rare autosomal recessive disease with a frequency estimated at around 1/100,000 births [1,2,3,4]. ABCB11/BSEP is a transporter of the ATP-binding cassette (ABC) family expressed at the canalicular membrane of hepatocytes, where it transports bile acids (BAs) into the canaliculi driving the BA-dependent bile flow [5]. In PFIC2, impaired biliary BA secretion leads to decreased bile flow, bile salt accumulation in hepatocytes, ongoing hepatocellular damage, and increased risk of hepatocellular carcinoma [1,2,6]. Clinical signs of cholestasis usually appear in the first months of life with jaundice and pruritus. In the liver of most children with PFIC2, ABCB11 is not detected at the canaliculus [1,2]. Patients develop fibrosis and end-stage liver disease before adulthood. In some patients, medical therapy with ursodeoxycholic acid (UDCA), rifampicin, inhibitors of the apical sodium-dependent BA transporter (ASBTi), and surgical biliary diversion may improve the medical condition or provide some symptomatic relief [1,7]. Nevertheless, in most cases, liver transplantation is required because of unremitting pruritus, hepatic failure, or hepatocellular carcinoma [1,6].

More than 1600 disease-causing *ABCB11* variations have been reported and listed in https://bravo.sph.umich.edu/, https://gnomad.broadinstitute.org/ and http://abcm2.hegelab.org/ (all accessed on 1 April 2025), mostly point variations (missense, nonsense, and splicing) located throughout the 27 exons [1,2]. Similar to class II variations of *ABCC7* encoding Cystic Fibrosis Transmembrane conductance Regulator (CFTR), the transporter involved in cystic fibrosis (CF), some *ABCB11* missense variations (E297G, D482G, T1210P) have been shown to result in the expression of immature proteins which accumulate in the endoplasmic reticulum (ER), where they are likely to undergo ER-associated degradation (ERAD), whereas they retain their transport function [8,9].

The field of personalized pharmacotherapy research is very active in diseases involving proteins of the family of ABC transporters, including hepatocellular genetic cholestasis, such as PFIC2, as well as CF for which very efficient compounds have been identified and are now used in routine clinical practice [9,10,11,12]. Previous in vitro studies have shown that sodium 4-phenylbutyrate (4-PB), a European Medicines Agency (EMA)- and Food Drug Administration (FDA)-approved butyrate analog used as an ammonia scavenger in patients with urea cycle disorders (UCDs), was able to increase cell surface/canalicular expression of some ABCB11 missense variants, including E297G, D404G, D482G, G982R, R1128C, T1210P and R1231Q [9,11,13,14,15], as well as of wild type (wt) ABCB11 [13,14,15]. 4-PB is a chemical chaperone able to correct the folding of ER-retained proteins. Hayashi et al. also showed in vitro that the increase in cell surface expression of some of these variants (E297G, D482G, R1231Q) resulted in an increase in BA transport [13,14,15]. Based on these preclinical data, we and others have treated selected patients with ABCB11-deficiency with 4-PB. Treatment with 4-PB of six patients with PFIC2 carrying the G982R, R1128C, T1210P, R1231Q, and the Y157C/G1298R variations of *ABCB11* and of one BRIC2 patient carrying the D404G variation resulted in a decrease in cholestasis [9,11,14,15,16].

Because of its formulation, treatment with 4-PB requires the patient to take up to 40 pills daily, which is at the origin of compliance issues [17,18]. The development of new 4-PB or non-4-PB related correcting drugs is necessary to achieve higher clinical benefit for patients harboring ABCB11 class II variations. Recently, another presentation of 4-PB has been clinically approved to treat patients with UCDs, namely glycerol phenylbutyrate (GPB, Ravicti^®^, https://www.ema.europa.eu/en/documents/variation-report/ravicti-h-c-3822-p46-0004-epar-assessment-report_en.pdf, accessed on 1 April 2025). As a triglyceride-related glycerol tri-ester, GPB acts as a prodrug of 4-PB. Structurally, it comprises three phenylbutyrate molecules covalently linked to the three alcohol functions of glycerol [19]. We reported the successful switch from 4-PB to GPB in a PFIC2 patient carrying the G982R variation [20]. Both 4-PB and GPB are transformed into successive intermediate metabolites, resulting in phenylacetate (PA), which is believed to be the active compound responsible for the beneficial clinical effects observed in patients with UCDs [19,21].

Besides 4-PB and GPB, other compounds (correctors) could have the potential to increase the canalicular expression of ABCB11 missense variants. Interesting correctors emerged from studies performed on ER-retained ABCC7/CFTR variants. These compounds included the VX-445 (elexacaftor) and VX-661 (tezacaftor), part of the clinically approved Trikafta^®^/Kaftrio^®^ tri-therapy for some patients with CF, as well as C18 (an investigational compound identified by high-throughput screening) [22], suberoyl anilide hydroxamic acid (SAHA, Vorinostat^®^) and curcumin. Lastly, previous studies suggested that UDCA, a clinically approved drug widely used in cholestatic patients, could also carry retargeting properties [11,23]. We previously showed in vitro that UDCA ± 4-PB increased the canalicular expression of the Abcb11^R1128C^ class II variant, among other Abcb11 variants, but the consequence of this treatment in terms of transport function was not assessed [11].

In the present study, we assessed the in vitro effect of 4-PB, GPB, 4-PB-analog, or homolog drugs and of non-4-PB-related chemical correctors (UDCA, SAHA, C18, VX-445, and VX-661) on the canalicular expression and the BA transport function of the Abcb11^R1128C^ variant. We showed that 4-PB, GPB, and PA, as well as two compounds with structural homology with 4-PB (3-hydroxy-2-methyl-4-phenylbutyrate, HMPB and 4-oxo-1,2,3,4-tetrahydro-naphthalene-2-carboxylic acid, OTNC) and UDCA, SAHA, C18, VX-445, and VX-661, increased the canalicular expression and the BA transport function of Abcb11^R1128C^.

## 2. Results

### 2.1. Three-Dimensional Structure Analysis Predicts a Folding Defect of the Abcb11^R1128C^ Variant

We have previously reported that the Abcb11^R1128C^ variant is retained in the ER, suggesting a misfolded variant protein [11]. To assess the mechanism underlying this defect observed in the R1128C variant, we analyzed the position and neighborhood of the Arg1128 residue within the 3D structure of ABCB11 (pdb: 8PMD) [24]. Arg1128 is localized in the nucleotide-binding domain 2 (NBD2), far from the NBD1/NBD2 interface and thus from ATP binding sites. However, it forms a salt bridge with Asp1131 (Figure 1). It thus contributes to the internal structure of this part of the NBD2. Moreover, Arg1128 is located in a contact zone, close to the intracellular loops (ICLs) from Transmembrane Domains TMD1 and TMD2 (with salt-bridge linking TMD1 R289 and TMD2 D848) and the segment linking TMD2 to NBD2 (in which I1061 forms contact). A variant of Arg1128 may induce a local destabilization of the protein that could directly alter the NBD2/TMD2 assembly, predicting a folding defect of the ABCB11^R1128C^ variant.

### 2.2. Effect of 4-Phenylbutyrate, Glycerol Phenylbutyrate and Phenylacetate on the Localization and the Function of the Abcb11^R1128C^ Variant

Twenty-four hours after transient transfection of the plasmid encoding the green fluorescent protein (GFP)-tagged Abcb11 in polarized hepatocellular Can 10 cells, the canalicular expression of the Abcb11^wt^ and Abcb11^R1128C^ proteins was assessed. As expected, Abcb11^wt^ localized exclusively at the bile canaliculi, identified by phase contrast and immunolocalization of ZO-1 (Figure 2A). In contrast, Abcb11^R1128C^ (treated with the vehicle PBS or DMSO) was retained intracellularly, as previously reported (Figure 2B) [11].

Quantification studies showed that treatment with 4-PB or GPB as well as incubation at 27 °C—the latter condition, which is not achievable in patients, being known to stabilize misfolded variant proteins in vitro [25]—partially increased the canalicular expression of Abcb11^R1128C^ (Figure 2B,C). Indeed, the proportion of Can 10 cells with Abcb11^R1128C^-GFP enrichment at the canalicular pole increased from 15% (PBS) to 60% and 96%, after treatment with 1 mM 4-PB, and incubation at 27 °C, respectively; and increased from 20% (DMSO) to 68%, after treatment with 1 mM GPB (Figure 2E). The activity of the Abcb11^R1128C^ missense variant was assessed in MDCK cells stably co-expressing Abcb11^R1128C^ and the basolateral BA transporter Na-taurocholate co-transporting polypeptide (Slc10A1, Ntcp) by measuring the vectorial transport of ^3^H-taurocholate ([^3^H]-TC) across MDCK monolayer cultured in insert. As expected, confocal studies showed that Abcb11^wt^ and Ntcp localized at the apical and the basolateral membranes of MDCK cells, respectively. Abcb11^R1128C^ localized in the cytoplasm of MDCK cells (Supplementary Figure S1). In addition, the transcellular transport of [^3^H]-TC measured in MDCK cells expressing Abcb11^R1128C^ and Ntcp was significantly lower than that measured in MDCK expressing Abcb11^wt^ and Ntcp (30% of the activity of the Abcb11^wt^ protein) and was comparable to that measured in MDCK cells expressing only Ntcp (28% of the Abcb11^wt^ protein, Figure 3A), suggesting that the mis-localization of Abcb11^R1128C^ resulted in the absence of BA transport. After 24 h of treatment with 4-PB (1 mM), GPB (1 mM), or growing cells at 27 °C, the transcellular transport of [^3^H]-TC in MDCK Abcb11^R1128C^ Ntcp increased significantly compared to controls, reaching 53%, 68% and 69% of the Abcb11^wt^ transport activity, respectively (Figure 3A).

We then assessed the effect of a lower dose of these drugs (concentration based on previous studies [26,27]) on the canalicular localization and the function of the ER-retained Abcb11^R1128C^ variant. In Can 10 cells, both 4-PB and GPB, at 150 µM, increased the canalicular expression of the R1128C variant to levels close to those observed with the 1 mM dose (49% and 60% with 4-PB and GPB, respectively) (Figure 2C,E). Interestingly, this lower dose of 150 µM led to approximately the same effect as the 1 mM dose on the transcellular transport of [^3^H]-TC in MDCK cells expressing Abcb11^R1128C^ and Ntcp (particularly with GPB), reaching 45% and 68% of the Abcb11^wt^ transport activity with 4-PB and GPB treatments, respectively (Figure 3A). GPB at the dose of 150 µM significantly restored the function of Abcb11^R1128C^ at a higher level than 4-PB, regardless of the concentration of 4-PB (Figure 3A). Treatment with PA (150 µM), the active metabolite of 4-PB and GPB (Figure 4) also increased the proportion of canalicular expression of Abcb11^R1128C^ to 66% (Figure 2E) and its transcellular transport of [^3^H]-TC to 59% of the Abcb11^wt^ transport activity (Figure 3A).

### 2.3. Identification of 4-Phenylbutyrate-Analogs or Homologs

Based on the common structural features displayed by 4-PB or PA, the BioCIS chemical library (a local compound collection contributor to The French National Compound Library-Chimiothèque Nationale-https://chembiofrance.cn.cnrs.fr/en/composante/chimiotheque, accessed on 10 January 2013) was screened in silico by pharmacophore homology. Two compounds with structural homology with 4-PB or PA were identified, namely methyl 3-hydroxy-2-methyl-4-phenylbutyric ester (MHMPB) and 4-oxo-1,2,3,4-tetrahydro-naphthalene-carboxylate (OTNC) (Figure 5). 

In addition, to simplify the chemical structures and identify structural determinants essential to the pharmacological activity, three derivatives of MHMPB have been synthesized: (i) 3-hydroxy-2-methyl-4-phenylbutyrate (HMPB) was produced by saponification of MHMPB [28] and (ii) the derivatives, methyl (*E*)-2-methyl-4-phenylbut-2-enoate (MMPB-2E) and methyl (*E*)-2-methyl-4-phenylbut-3-enoate (MMPB-3E) were prepared by dehydration of MHMPB [29] (Figure 5). These compounds are structural analogs of 4-PB preserving the pharmacophore elements, a phenyl ring separated from a carboxyl group by a flexible or semi-rigid three-carbon chain (Figure 4 and Figure 5). 4-PB is metabolized into PA through a cycle of chain shortening (Figure 4). Kasumov et al. identified the presence of the β-hydroxy phenylbutyrate (HPB) metabolite in the urine of patients treated with 4-PB, allowing them to decipher the metabolic pathway of 4-PB into PA [21]. The latter proceeds by the subsequent formation of 4-phenylcrotonate, HPB, and then β-keto phenylbutyrate [21] (Figure 4). High structural homology between 4-phenylcrotonate and MMPB-2E, as well as between HPB and HMPB (or the prodrug MHMPB), suggests that the synthetic compounds HMPB, MHMPB, MMPB-2E and MMPB-3E may follow a similar metabolic pathway resulting in the production of PA, the active metabolite (Figure 4 and Figure 5). The HPB-like structure of HMPB and MHMPB suggests that these compounds could be metabolized into PA more rapidly than 4-PB and are probably more easily absorbed thanks to higher lipophilicity than 4-PB or HPB. OTNC is a constrained PA analog, presenting a suppression of the conformational freedom of the carboxylic arm (Figure 5). The non-enzymatic curcumin hydrolysis produces ferulic acid and dihydro-ferulic acid [30](Figure 4). Ferulic acid shows structural homology with 4-phenylcrotonate and MMPB-2E and could be taken up by the same metabolic pathways as 4-PB to form a metabolite analog.

### 2.4. Effect of the 4-Phenylbutyrate-Analogs or Homologs on the Localization and the Function of the Abcb11^R1128C^ Variant

The effects of the five 4-PB-analogs—MHMPB, HMPB, MMPB-2E, MMPB-3E, and OTNC—were studied at the concentration of 150 µM. In Can 10 cells, treatment with MHMPB and curcumin (1 µM) tended to increase the canalicular expression of Abcb11^R1128C^ but by less than 150 µM 4-PB (42% and 34% vs. 49%, respectively) (Figure 2C,E). HMPB and OTNC increased the proportion of canalicular expression of Abcb11^R1128C^ at a slightly higher level than 4-PB (150 µM) (54% and 55% vs. 49%, respectively) (Figure 2C,E). MMPB-2E and MMPB-3E had no effect (Figure 2C,E) on the traffic of the Abcb11^R1128C^ variant. Among the five 4-PB-analogs and curcumin, only HMPB and OTNC restored canalicular expression of Abcb11^R1128C^ at a higher level than 4-PB (150 µM) and were tested for their effect on the transport activity in MDCK cells expressing Abcb11^R1128C^ and Ntcp. HMPB and OTNC significantly increased the transcellular transport of [^3^H]-TC by the Abcb11^R1128C^ variant at a level close to that of 150 µM 4-PB (46% and 42%, respectively vs. 45% of the Abcb11^wt^ transport activity) (Figure 3A).

### 2.5. Effect of UDCA and of Other Non-4-PB Related Chemical Correctors on the Localization and the Function of the Abcb11^R1128C^ Variant

Here, we confirm our previous results that UDCA (50 µM) ± 4-PB (1 mM) increased the canalicular expression level of the Abcb11^R1128C^ variant in Can 10 polarized cells [11] and at a higher level than the one of 150 µM 4-PB (64% and 79%, respectively, for UDCA and UDCA + 4-PB vs. 49% for 4-PB alone) (Figure 2D,F). Moreover, we demonstrated in transport activity assays that UDCA significantly increased [^3^H]-TC transcellular transport through Abcb11^R1128C^ at the same level as with 150 µM 4-PB (46% vs. 45%, respectively) (Figure 3B). Interestingly, a combined treatment with UDCA and 4-PB further increased the activity of the Abcb11^R1128C^ variant to 63% of the Abcb11^wt^ transport activity, suggesting an additive effect between these compounds (Figure 3B). We have also analyzed other non-4-PB-related chemical drugs identified as CFTR correctors. In Can 10 cells, SAHA (1 µM) and C18 (3 µM) increased the canalicular expression of Abcb11^R1128C^ at a significantly higher level than 150 µM 4-PB (61% and 67% for SAHA and C18, respectively vs. 49% for 4-PB alone) (Figure 2D,F). In our in vitro transcellular transport system, those drugs increased the [^3^H]-TC transcellular transport of Abcb11^R1128C^ at a level similar to that of 4-PB (55% and 48% for SAHA and C18, respectively vs. 45% of the Abcb11^wt^ for 150 µM 4-PB alone) (Figure 3B). In addition, we studied the canalicular localization of the Abcb11^R1128C^ in HepG2 cells, a well-characterized hepatocellular polarized line of human origin. In HepG2 cells, as in Can 10 cells, we showed that the Abcb11^R1128C^ variant was retained in the cytoplasm while Abcb11^wt^ was expressed exclusively at the canalicular membrane of the cells, immunolabeled using anti-ABCC2 antibodies (Figure 6A). After 24 h of treatment, we analyzed the effects of both Vertex Pharmaceuticals correctors VX-445 (0.5 µM) and VX-661 (1 µM). Immunolabeling analyses (Figure 6A) and related quantification showed that VX-445, VX-661, and a combination of both drugs increased the percentage of transfected cells expressing Abcb11^R1128C^ at the canalicular membrane from 4% to 20%, 30%, and 34%, respectively (Figure 6B). We also assessed the effect of these drugs on the [^3^H]-TC transport activity of Abcb11^R1128C^ in MDCK-Abcb11^R1128C^ Ntcp cells (Figure 3B). Treatment with VX-445, VX-661, and a combination of both drugs increased the transport activity of the Abcb11^R1128C^ variant from 31% to 53%, 55%, and 74% of the [^3^H]-TC transport activity of the Abcb11^wt^ protein, respectively. Interestingly, the VX-445/VX-661 combination was particularly effective and significantly exceeded the level of transport activity obtained after treatment with 4-PB (150 µM).

## 3. Discussion

Herein, we further characterized the Abcb11^R1128C^ missense variant and assessed the ability of various chemical correctors to rescue its canalicular expression and activity. We have previously shown that the defective ABCB11^R1128C^ variant identified in PFIC2 patients resulted in the synthesis of a protein retained in the ER and that treatment with the non-protein-specific chemical chaperone 4-PB (EMA- and FDA-approved for UCDs) partially corrected the targeting of Abcb11^R1128C^ variant to the canalicular membrane both in vitro and in one PFIC2 patient [11]. In this patient, treatment with 4-PB led to a decrease in clinical and biological parameters of cholestasis, suggesting that this variant retains a transport function, despite its folding/traffic defect [11]. The 3D structure analysis performed in this study predicted a folding defect of the Abcb11^R1128C^ variant, which could explain its retention in the ER. This amino acid variation could affect both the NBD2 folding/stability and interdomain association without disturbing the ATP binding and, thus, the function of the retargeted protein. In line with this in silico prediction, we showed herein that the trafficking defect of the Abcb11^R1128C^ variant resulted in a total absence of BA transport activity in MDCK cells and that treatment with 4-PB increased both the canalicular expression and the BA transport activity of the Abcb11^R1128C^ variant. These findings suggested that the Abcb11^R1128C^ variant belongs to class II genetic variations (i.e., variations leading to a trafficking defect without altering the function of the protein) [31,32] and supported the need to identify drugs (correctors) able to induce the delivery of Abcb11^R1128C^ to the cell surface. Herein, we investigated 4-PB derivatives and several CFTR correctors as potential correctors of the Abcb11^R1128C^ variant.

GPB, another EMA- and FDA-approved drug to treat UCDs, which is composed of three PB molecules, increased the canalicular expression and the activity of the Abcb11^R1128C^ variant to a level close to 70% of the wild type protein. These increases in canalicular expression and in activity were significantly higher than those observed with 4-PB used at the same doses. Such superiority of the GPB over the 4-PB might be due to an intrinsic trafficking rescue effect of glycerol previously reported for the misfolded variant protein ΔF508-CFTR [33] and other Abcb11 missense variants [34]. The in vitro effects of GPB reported here are important, as GPB has no sodium burden and offers palatability and pharmacokinetic advantages over 4-PB [19]. Indeed, GPB is an uncharged and more lipophilic compound than 4-PB, allowing GPB to get through barriers more easily [35]. These results, together with our previous report on the successful switch from 4-PB to GPB in a PFIC2 patient carrying the ABCB11^G982R^ variant, support the off-label use of GPB as an alternative to 4-PB in selected PFIC2 patients [20]. The effects of treatment with PA observed in this study are in line with previous data suggesting that 4-PB (and GPB) may be considered as prodrugs and that their correcting effect is related to the production of the PA active moiety through β-oxidation [21].

The significant efficiency of OTNC, 4-PB, and PA—which are all carboxylate derivatives—in correcting the traffic supports the importance of the carboxylic function for the chaperone activity. OTNC may be active without prior metabolization. In contrast, the MHMPB homolog that has a methyl ester function instead of a carboxylate group may require prior hydrolysis to become pharmacologically active (Figure 5), suggesting its lower efficiency in readdressing the Abcb11^R1128C^ to the canaliculus. MHMPB can be considered as a prodrug of HMPB, MMPB-2E and MMPB-3E. HMPB could be more active than MHMPB, MMPB-2E, and MMPB-3E thanks to the presence of its carboxylic function. The presence of the Michael acceptor function in MMPB-3E may further decrease its efficacy, even inducing toxicity as an alkylating agent [36]. Similarly, by tautomerization, MMPB-2E may be transformed into MMPB-3E, likely to explain its non-efficacy and cytotoxicity.

Medicinal chemistry studies have to be pursued to find new efficient 4-PB metabolism activators to promote the release of PA and optimized for class II ABCB11 variations. Particular attention should be paid to the presence of a carboxylic function, which seems to play a role in the chaperone activity of PB-like drugs.

Our results show that non-4-PB-related compounds could also be of interest in increasing the canalicular expression and function of the variant Abcb11^R1128C^. UDCA, a widely used «anti-cholestatic» drug, has been shown both in vitro and in PFIC2 patients to increase canalicular expression of several ER-retained ABCB11 variants, including Abcb11^R1128C^ [11,23,37]. In this study, we showed that UDCA treatment also increased the activity of the Abcb11^R1128C^ protein in MDCK cells. In addition to the mechanisms already identified and promoting the insertion and stabilization of ABCB11 in the canalicular membrane of hepatocytes by UDCA [38], the presence of the carboxyl group may be one pharmacophoric element important for its chaperone activity. All together these data suggest that UDCA, besides its usual non-specific “anti-cholestatic” properties, also displays a corrector effect that may account for its clinical benefit observed in PFIC2 patients harboring at least one missense variation [1]. The significant additive effect of UDCA and 4-PB observed in our in vitro models supports the use of the combination of these drugs in selected PFIC2 patients carrying an ABCB11 ER-retained variant, as previously reported [11].

In this study, we also evaluated CFTR correctors. Over the past few years, the discovery and subsequent development of CFTR modulators (such as potentiators and correctors) have revolutionized the standard of care for CF patients. The ability of SAHA to readdress Abcb11^R1128C^ to the canaliculus, partially restoring its activity, confirms its potential to correct misfolded proteins, as supported by several previous studies [39,40,41]. Bodas et al. demonstrated that SAHA induces ΔF508-CFTR trafficking by inhibiting its proteasomal degradation [41]. This mechanism may account for the effect observed in our study, as SAHA has a bio-isostere carboxylate function. SAHA is a synthetic hydroxamic acid derivative structurally close to 4-PB (Figure 5) without preserving the pharmacophore elements (neither a carbon chain nor a carboxylate), suggesting that SAHA does not follow the same metabolic pathways as 4-PB and has a different mechanism of action. Some CFTR correctors are also clinically approved for selected variations in CF patients. The three compounds developed by Vertex Pharmaceuticals C18 (formerly VRT-534), VX-445 (elexacaftor), and VX-661 (tezacaftor) were all effective at a low dose to readdress Abcb11^R1128C^ protein to the canalicular membrane of Can 10 or HepG2 cells and allowed a significant improvement of the activity of Abcb11^R1128C^ in MDCK cells. The small-molecule compound C18 (considered a first-generation corrector) has not advanced to clinical trials like other more efficient CFTR modulators such as VX-809 (lumacaftor) [42] approved in combination with the potentiator VX-770 (Ivacaftor) (Orkambi^®^). However, its structure has influenced the design of more effective correctors such as VX-661 with improved binding affinity and pharmacokinetics. Indeed, VX-661 and C18 share a common scaffold (pharmacophore) [43]. In our study, C18 allowed only a 3.5-fold increase of the basal level of the canalicular Abcb11 ^R1128C^ protein versus a 9-fold increase induced by VX-661, supporting a better efficacy of VX-661. VX-445 is a next-generation CFTR corrector with a mechanism of action different from the first-generation corrector VX-661 [31]. A combination of VX-445/VX-661 compounds showed a very interesting additive effect in rescuing the traffic and the function of Abcb11^R1128C^. As previously shown, while both correctors promote proper folding, they bind to different sites on the CFTR protein, enhancing its structural stability more effectively than either alone [43,44,45]. VX-661 binds and stabilizes the transmembrane domain TMD1 at an early stage of CFTR biogenesis (type I corrector) [46,47]. VX-445 stabilizes TM10 and TM11, the two domain-swapped helices of TMD2 forming the intracellular loop ICL4, which interacts with NBD1 (type III corrector) [48]. VX-445 thereby strengthens the TMD/NBD1 interface and allows the formation of a protease-resistant form. It would be interesting to know if these compounds act similarly in ABCB11 than in CFTR and if they share similar binding sites. Despite belonging to the same superfamily, sequence and structural features differ between ABCBs and ABCCs (e.g., absence in ABCB transporters of an ABCC-specific lasso, which alters the characteristics of the hydrophobic pocket at the base of TMD1 [49] and of the pocket involving TM10 and TM11). Further studies are thus needed to explore the specific mechanisms of action of these correctors on the ABCB family. The clinically approved triple combinatorial therapy composed of VX-661, VX-445 and the potentiator ivacaftor (VX-770) constitutes the most effective modulator therapy nowadays for patients with CF [50]. The combination of potentiators and correctors has to be explored to try to further improve the activity of the retargeted ABCB11 class II variants, all the more so as we have previously shown that CFTR potentiators, such as ivacaftor, were able to rescue BA secretion activity due to selected *ABCB11* missense variations [32,51].

Our in vitro studies provide proof-of-concept that several 4-PB-related and non-4-PB related drugs have the potential to correct selected ER-retained *ABCB11* missense variations. However, further investigations are required to analyze the functional effect of these drugs on BA transport activity including time courses studies and evaluation of kinetic parameters (V_max/K_m). In vitro studies using patient-derived hepatocytes or organoids as well as in vivo studies in animal models should also be considered to further support the potential of these drugs in the clinical setting. From this viewpoint, the use of a non-tagged ABCB11 plasmid should also be considered. Finally, therapeutic windows and off-target effects of these molecules should be evaluated both in vitro and in vivo in the frame of ADME-Tox (Administration, Disposition, Metabolism, Elimination, Toxicity) analyses.

The present in vitro study enabled the further characterization of an ER-retained ABCB11 missense variant and the identification of new correcting drugs able to improve the trafficking and function defects caused by a class II *ABCB11* variation. These molecules, alone or in combination, increased transporter activity to levels between 45% and 75% of the wt protein activity, thus providing a real hope of correcting the disease phenotype. Our data provides experimental evidence that GPB and CFTR correctors may offer new “off-label” therapeutic options for selected patients with ABCB11 deficiency caused by class II variations, affecting the intracellular traffic of the transporter.

## 4. Materials and Methods

### 4.1. Chemistry Materials

Commercially available reagents were used throughout without further purification other than those detailed below. Before use, tetrahydrofurane (THF) was dried using a solvent purifier system. All anhydrous reactions were carried out under an argon atmosphere. Analytical thin layer chromatography was performed on 60F-254 precoated silica (0.2 mm) on glass and was revealed by UV light and *p*-anisaldehyde staining. Flash chromatography separations were carried out on silica gel (40–63 µm). Infrared (IR) spectra were obtained as neat films. ^1^H and ^13^C nuclear magnetic resonance (NMR) spectra were recorded respectively at 300 or 400 MHz and 75 or 100 MHz unless otherwise specified. The chemical shifts for ^1^H NMR were recorded in ppm downfield from tetramethylsilane (TMS) with the deuterated solvent resonance as the internal standard. Coupling constants (*J*) are reported in Hz and refer to apparent peak multiplications. High-resolution mass spectrometry (HRMS) (ElectroSpray Ionization, ESI) analyses were performed with a time-of-flight mass spectrometer yielded ion mass/charge (*m*/*z*) ratios in atomic mass units. The atmospheric pressure chemical ionization (APCI) mass spectra were recorded on a quadrupole time-of-flight 6546 Agilent Technologies mass spectrometer (https://www.agilent.com/). The purity of synthesized compounds was determined by reverse phase HPLC using a 150 mm × 2.1 mm (3.5 μm) C18-column: compounds were eluted over 20 min with a gradient from 95% ACN/5% water/0.2% FA to 5% ACN/95% water/0.2% FA. All compounds were purified to >95% purity as determined by HPLC. OTNC and MHMPB were synthesized according to the procedure described in the literature; their physical data are congruent with those published [52,53].

### 4.2. Chemical Synthetic Procedures

#### 4.2.1. The Methyl 3-Hydroxy-2-methyl-4-phenyl Butyric Ester (MHMPB)

To a solution of LDA freshly prepared from di-isopropyl-amine (4.14 g, 40.9 mmol) and 28.5 mL of n-BuLi (1.44 mmol/mL hexanes) in 100 mL of THF, is added dropwise methyl propionate (3.9 mL, 3.60 g, 40.8 mmol) at −78 °C. The mixture is stirred for 30 min. Then, phenyl ethanal (2.45 g, 20.4 mmol) is rapidly added at −78 °C. After 30 min of stirring, the reaction mixture is allowed to warm at −35 °C and the reaction is quenched by the sequential addition of 15 mL of aqueous THF (1:1 *v*/*v*) and 15 mL of a saturated NH_4_Cl aqueous solution. The solution mixture is neutralized with concentrated HCl and extracted twice with diethyl ether (2 × 100 mL). The combined organic layers are washed with a 5% HCl solution (2 × 50 mL), water (50 mL) and a saturated NaCl solution (50 mL), dried over MgSO_4_ and concentrated under vacuum to afford a crude oil (4.60 g). Distillation at 102 °C/1 Torr yielded 3.2 g (75%) of 3:1 diastereomeric mixture of MHMPB, IR (neat, cm^−1^): 3510, 3425, 2980, 1735, 1270, 1140, 700; ^1^H NMR (CDCl_3_, 300 MHz) δ 7.35–7.22 (m, 5H_Ar_),4.21–3.90 (m, CH-O), 3.70 (s, 3H, OCH_3_), 2.7–2.2 (m, 1H, CH-CO),2.31 (br s, 1H, OH), 2.93–2.53 (m, 2H, CH_2_), 1.23 (d, *J* = 7.3 Hz, 3H, CH_3_); ^13^C NMR (CDCl_3_, 75 MHz) δ 176.3, 175.9 (CO), 138.3, 138.2 (C_Ar_), 129.5, 129.3 (2 × CH_Ar_), 128.4 (2 × CH_Ar_), 126.4 (CH_Ar_), 74.3, 73.0 (CH_3_O), 51.7 (CH-O), 44.5, 44.0 (CH), 41.0, 40.6 (CH_2_), 14.1, 12,1 (CH_3_); Anal. calcd. for C_12_H_16_O_3_: C 69.21, H 7.74; found C, 69.31, H 7.70.

#### 4.2.2. The 3-Hydroxy-2-methyl-4-phenylbutyrate (HMPB)

To a solution of MHMPB (278 mg, 1.3 mmol) in aqueous THF (3.7 mL; 1:1 *v*/*v*), lithium hydroxide (32 mg, 1.3 mmol) is added at room temperature and the mixture is stirred for 12 h. The aqueous phase was washed with ethyl acetate (5 mL), and the aqueous phase was acidified at 0 °C with a 1N HCl aqueous solution. The precipitate is filtered and recrystallized in acetone/hexane to afford HMPB as white crystals (114 mg, 44% yield). The 100% purity of HMPB (t_R_ = 10.88 min) was determined by reverse phase HPLC (λ = 254 nm). ^1^H NMR (300 MHz, acetone-*d*_6_): *δ* 10.68 (br s, 1H), 7.28–7.17 (m, 5H), 4.16 (dt, *J* = 7.7, 5.0 Hz, 1H), 3.85 (br s, 1H), 2.83 (dd, *J* = 13.6, 5.2 Hz, 1H), 2.74 (dd, *J* = 13.6, 8.1 Hz, 1H), 2.48 (qd, *J* = 7.1, 5.2 Hz, 1H), 1.23 (d, *J* = 7.1 Hz, 1H); ^13^C NMR (75 MHz, acetone-*d*_6_): δ 176.7 (C=O), 140.4 (C_Ar_), 130.3 (2 CH_Ar_), 129.1 (2 CH_Ar_), 126.9 (CH_Ar_), 73.9 (CH-O), 45.2 (CH-CO), 42.1 (CH_2_), 11.7 (CH_3_); HRMS (*m*/*z*): [M + H]^+^ calcd for C_11_H_15_O_3_, 195.1016; found 195.1067.

#### 4.2.3. General Procedure for Methyl (E)-2-Methyl-4-phenylbut-3-enoate (MMPB-3E) and Methyl (E)-2-Methyl-4-phenylbut-2-enoate (MMPB-2E)

To a solution of MHMPB (194 mg, 1 mmol) and DMAP (244 mg, 2 mmol) in THF (2 mL, 0.4 M) was added 4-nitrobenzenesulfonyl chloride (222 mg, 1 mmol) at room temperature. The mixture was heated at 40 °C for 2h. The crude is extracted with ethyl acetate and washed successively with a 1 N HCl aqueous solution, a saturated NaHCO_3_ aqueous solution then brine. The organic phase was dried over MgSO_4_ and concentrated under vacuum to afford an oily liquid (177 mg, 93% yield) with, respectively, a ratio of (MMPB-3E/MMPB-2E) (7/3). Both isomers were separated on a Xbridge column with an isocratic eluent water/methanol 4/6 and fully characterized. Physical data of MMPB-3E are in accordance with the literature [54].

MMPB-3E: ^1^H NMR (400 MHz, CD_3_OD): *δ* 7.24–7.37 (m, 5 H_Ar_), 6.48 (d, *J* = 16.2 Hz, 1 H), 6.28 (dd, *J* = 16.2, 7.1 Hz, 1 H), 3.71 (s, 3H), 3.33 (m, 1 H), 1.37 (d, *J* = 1.1 Hz, 3 H); ^13^C NMR (100 MHz, CD_3_OD): *δ* 175.1 (C=O), 136.8 (C_Ar_), 131.2 (=CH), 128.6 (2 × CH_Ar_), 128.5 (2 × CH_Ar_), 127.5 (CH_Ar_), 126.3 (=CH), 51.9 (OCH_3_), 43.1 (CH), 17.4 (CH_3_); HRMS (*m*/*z*): [M + H]^+^ calcd for C_12_H_14_O_2_, 191.1067; found 191.1116.

MMPB-2E: ^1^H NMR (400 MHz, CD_3_OD): *δ* 7.18–7.31 (m, 5 H_Ar_), 6.93 (dt, *J* = 7.2, 1.4 Hz, 1 H), 3.74 (s, 3 H), 3.54 (d, *J* = 7.2 Hz, 2 H), 1.97 (d, *J* = 1.4 Hz, 3 H); ^13^C NMR (100 MHz, CD_3_OD): *δ* 168.5(C=O), 140.4 (C_Ar_), 139.0 (2 × CH_Ar_), 128.5 (2 × CH_Ar_), 128.2 (CH_Ar_), 127.3 (=C), 126.4 (=CH), 51.8 (OCH_3_), 34.9 (CH_2_), 12.5 (CH_3_); HRMS (*m*/*z*): [M + H]^+^ calcd for C_12_H_14_O_2_, 191.1067; found 191.1118.

### 4.3. Three-Dimensional (3D) Structure Analysis

The cryo-EM 3D structure of human ABCB11 in nano-discs was considered for analyzing the R1128 position (catalytically inactivated ABCB11 variant (E1244Q) in complex with ATP, pdb 8PMD) [24]. Three-dimensional structure coordinates were manipulated and visualized using UCSF Chimera 1.13.1, which is a program for the interactive visualization and analysis of molecular structures and related data, including density maps, trajectories, and sequence alignments [55]. Inskape 0.92 was used for annotations in the Figure 1.

### 4.4. DNA Constructs and Mutagenesis

The C-terminus GFP vectors encoding wild type (wt) and missense R1128C variant of Abcb11 have been described before, as well as the C-terminus cMyc vector encoding Ntcp [11,51].

### 4.5. Transfection, Treatment and Quantification of Cells with Abcb11 Localized at the Canalicular Membrane

Can 10 and HepG2 cells, well-characterized hepatocellular polarized lines of rat and human origins, respectively, form pseudo-bile canaliculi in culture and were used to study the subcellular localization of Abcb11 wt and R1128C proteins. Vectors encoding GFP-tagged Abcb11 (wt and R1128C) were transiently transfected in Can 10 and HepG2 cells as previously published [3,11]. Six hours after transfection, Can 10 cells were either cultured at 27 °C or treated for 24 h at 37 °C as follows: 4-PB (1 mM or 150 µM), PA (150 µM; Sigma-Aldrich, St Quentin Fallavier, France) or vehicle (PBS); GPB (1 mM or 150 µM; MedChemExpress, Clinisciences, Montrouge, France), OTNC, MHMPB, HMPB, MMPB-2E and MMPB-3E (150 µM), UDCA (50 µM), SAHA, curcumin (1 µM; Sigma-Aldrich), C18 (3 µM; obtained from the Cystic Fibrosis Foundation Therapeutics panel library; https://www.cff.org/) or DMSO as a control vehicle at the same dilution (0.1% DMSO for all conditions). HepG2 cells were treated for 24 h with elexacaftor (VX-445; 0.5 µM) and/or tezacaftor (VX-661; 1 µM) or vehicle (DMSO, 0.1% final concentration).

Then, cells were fixed and immunolabeled using the following primary antibodies: mouse monoclonal anti-GFP (clones 7.1 and 13.1; Roche Diagnostics, Mannheim, DE, USA) and rat anti-zonula occludens 1 (ZO-1) [11] for Can 10 cells; mouse monoclonal anti-ABCC2 (clone M2I-4; Enzo Life Sciences, Villeurbanne, France) and rabbit polyclonal anti-GFP (ab290; Abcam, Cambridge, UK) for HepG2 cells. The appropriate Alexa-conjugated secondary antibodies (Molecular Probes/Thermo Fisher Scientific, Illkirch, France) were used at a 1:500 dilution [11]. Cells expressing Abcb11-GFP (wt or R1128C) forming bile canaliculi were examined by epifluorescence or confocal microscopy as previously described [11,32]. In Can 10 cells, bile canaliculi were identified by phase contrast and immunolocalization of ZO-1 or by immunolocalization of ABCC2 in HepG2 cells. Abcb11-GFP levels were quantified on epifluorescence sections using ImageJ software, version 1.54d (National Institutes of Health, Bethesda, MD, USA). Quantification of cells with Abcb11-GFP enrichment at the canalicular pole has been performed by measuring the percentage of Abcb11-GFP-positive cells expressing Abcb11 at the canalicular membrane [11,32].

### 4.6. Generation of MDCK Clones Stably Expressing Abcb11 and Ntcp

MDCK cells, a well-characterized polarized kidney cell line allowing vectorial transport, were stably transfected with Abcb11-GFP (wt or R1128C). To allow BA entry in MDCK cells, MDCK clones with the highest Abcb11-GFP (wt, R1128C) expression and parental MDCK cells, were infected with Ntcp-cMyc -encoding lentiviral particles as described [3]. Expression and localization of Abcb11-GFP and Ntcp-cMyc were analyzed by immunofluorescence using the following primary antibodies: rat monoclonal anti-cMyc (Clone JAC6; GeneTex, Irvine, CA, USA) and rabbit polyclonal anti-GFP (ab290; Abcam, Cambridge, UK), as previously described [32].

### 4.7. Taurocholate Transport Assay

MDCK clones stably expressing Abcb11-GFP (wt or R1128C) and/or Ntcp-cMyc were grown on membrane inserts as previously described [3]. The integrity of cell monolayers was assessed by transepithelial electrical resistance measurements and Lucifer yellow permeability tests, during the following day and before performing functional assays, as published [3,51]. The cell monolayers were treated for 24 h at 37 °C with 4-PB or GPB (1 mM or 150 µM), PA, HMPB, OTNC (150 µM), UDCA (50 µM), SAHA (1 µM), C18 (3 µM), VX-445 (0.5 µM), VX-661 (1 µM), vehicle (PBS or DMSO) or grown at 27 °C. Thereafter, the culture medium was replaced by prewarmed transport buffer in apical and basal compartments in the presence of tritium labeled taurocholate ([3H]-TC, Perkin Elmer, Waltham, MA, USA) in the basal compartment. After two hours, transcellular transport of [3H]-TC was calculated from the radioactivity in the apical compartment and normalized to the protein amount, as previously described [3].

### 4.8. Statistical Analyses

Data were analyzed using Prism v7 (GraphPad software, la Jolla, CA, USA) and expressed as means ± standard error of the mean (SEM). Statistical analyses were performed using one-way ANOVA, with a *p* value < 0.05 being considered statistically significant.

## Figures and Tables

**Figure 1:**
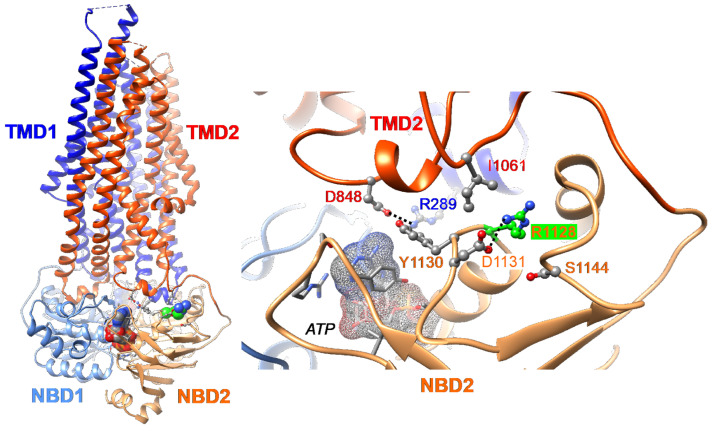
Three-Dimensional (3D) structure of human ABCB11 (E1244Q variant, in complex with ATP, pdb 8PMD [24]) visualized using Chimera. Ribbons are colored according to the considered domain (TMD1, dark blue; NBD1, blue; TMD2, red; NBD2, orange), while atoms are colored as follows (C, grey; O, red; N, blue), except for the arginine at position 1128 (R1128) (C atoms colored in green) (**left**). ATP is located at the interface between the NBD1 and NBD2 (**right**). R1128 forms a salt bridge with Asp1131 (D1131), contributing to the architecture of the NBD2 (colored in orange). This contact area is close to the intracellular loop (ICL2; in blue) and ICL3 (in red)—i.e., salt bridge between R289 and D848. The NBD1 is shown in blue. NBD, nucleotide-binding domain; TMD, transmembrane domain.

**Figure 2:**
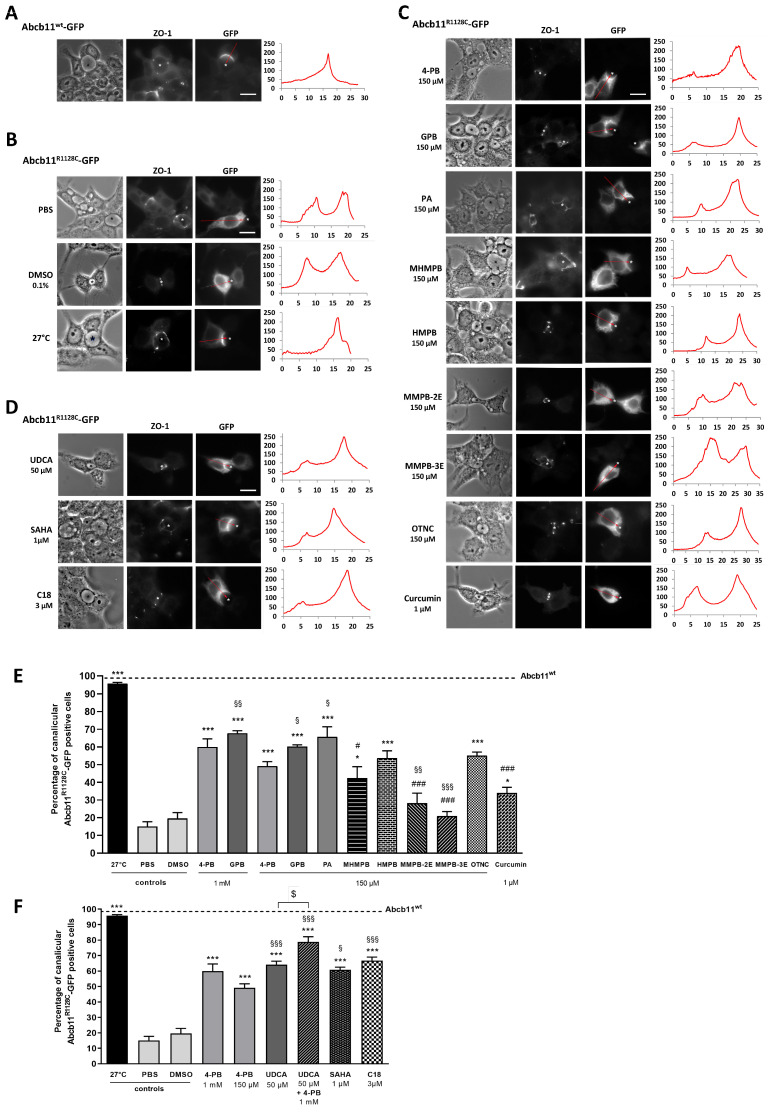
Effect of 4-PB analogs and non-analogs on the canalicular expression of Abcb11^R1128C^-GFP in hepatic polarized Can 10 cells. Cells were transiently transfected with Abcb11-GFP-encoding plasmids (wt or R1128C) and cultured at 27 °C or treated for 24 h at 37 °C with the indicated drugs and concentrations. (**A**–**D**) Immunolocalization of Abcb11-GFP (**right panels**) observed by epifluorescence microscopy. Phase contrast of cells (**left panel**) and tight junction protein zonula occludens 1 (ZO-1; **middle panel**) immunostaining revealed canalicular joint (white dots). Abcb11-GFP levels were quantified from left to right along the red arrow indicated on the corresponding image. The resulting graphs are shown on the right and represent the Abcb11-GFP fluorescence intensity (arbitrary unit) along the arrow axis (µm). Stars indicate canalicular structures. Bars: 10 µm. (**E**,**F**) Quantification of the effect of 4-PB analogs (**E**) and 4-PB non-analogs (**F**) on the canalicular expression of Abcb11^R1128C^-GFP. Among Abcb11-GFP positive cells forming canaliculi, the percentage of cells with Abcb11-GFP enrichment at the canalicular membrane was determined from at least 3 independent experiments per condition. The dashed line indicates the percentage of canalicular Abcb11-GFP positive cells of the wild type protein (Abcb11^wt^-GFP). * *p* < 0.05 and *** *p* < 0.001 vs. vehicle (PBS or DMSO); # *p* < 0.05 and ### *p* < 0.001 vs. 4-PB 1 mM treated cells; § *p* < 0.05, §§ *p* < 0.01 and §§§ *p* < 0.001 vs. 4-PB 150 µM; $ *p* < 0.05. 4-PB, 4-phenylbutyrate; GPB, glycerol phenylbutyrate; HMPB, 3-hydroxy-2-methyl-4-phenylbutyrate; MHMPB, methyl 3-hydroxy-2-methyl-4-phenyl butyric ester; MMPB-2E, methyl (E)-2-methyl-4-phenylbut-2-enoic ester; MMPB-3E, methyl (E)-2-methyl-4-phenylbut-3-enoic ester; OTNC, 4-oxo-1,2,3,4-tetrahydro-naphthalene-2-carboxylate; PA, phenylacetate; SAHA, suberoylanilide hydroxamic acid; UDCA: Ursodeoxycholic acid.

**Figure 3:**
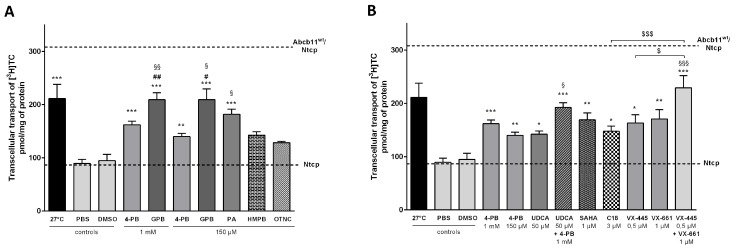
Some 4-PB analogs and non-analogs allow partial BA secretion in MDCK cells expressing the Abcb11^R1128C^ variant. MDCK clones stably expressing Abcb11^wt or R1128C^-GFP and/or Ntcp-cMyc were incubated at 27 °C or treated with 4-PB analogs (**A**) and 4-PB non-analogs (**B**) or vehicle (PBS or DMSO) for 24 h and transcellular transport of [^3^H]-taurocholate (TC) was measured. The upper and lower dashed lines indicate [^3^H]-TC transport measured in MDCK cells expressing both Abcb11^wt^ and Ntcp or Ntcp only, respectively. Means of at least five independent experiments for each tested condition are expressed as the amount of [^3^H]-TC, normalized to protein amount in each insert. * *p* < 0.05, ** *p* < 0.01 and *** *p* < 0.001 vs. vehicle (PBS or DMSO); # *p* < 0.05, ## *p* < 0.01 vs. 4-PB 1 mM treated cells; § *p* < 0.05, §§ *p* < 0.01 and §§§ *p* < 0.001 vs. 4-PB 150 µM treated cells; $ *p* < 0.05 and $$$ *p* < 0.001. 4-PB: 4-phenylbutyrate; GPB: glycerol phenylbutyrate; HMPB: 3-hydroxy-2-methyl-4-phenylbutyrate; OTNC: 4-oxo-1,2,3,4-tetrahydro-naphthalene-2-carboxylic acid; PA: phenylacetate; SAHA: suberoyl anilide hydroxamic acid; [^3^H]-TC: [^3^H]-taurocholate; UDCA: ursodeoxycholic acid.

**Figure 4:**
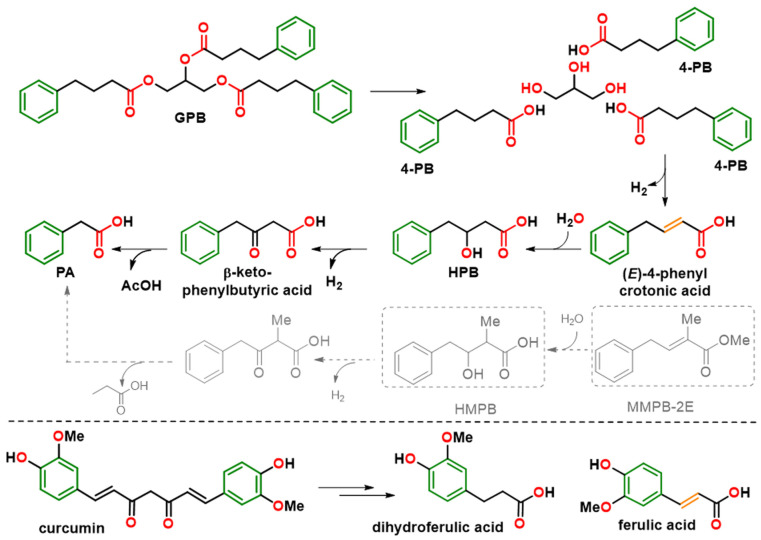
Metabolic pathways of GPB, 4-PB, and curcumin. Structural homology of synthetic compounds HMPB and MMPB-2E to the formed metabolites HPB and ferulic and crotonic acids, respectively, is shown. The colors of the molecule highlight the common pharmacophore elements: phenyl (green), alkyl chain (black), unsaturation (orange) and carboxylic group (red). The metabolic pathway that MMPB-2E could follow, by analogy with that of 4-PB, is represented in grey. 4-PB: 4-phenylbutyrate; GPB: glycerol phenylbutyrate; HMPB: 3-hydroxy-2-methyl-4-phenylbutyrate; HPB: β-hydroxy phenylbutyrate; MMPB-2E: methyl (E)-2-methyl-4-phenylbut-2-enoic ester.

**Figure 5:**
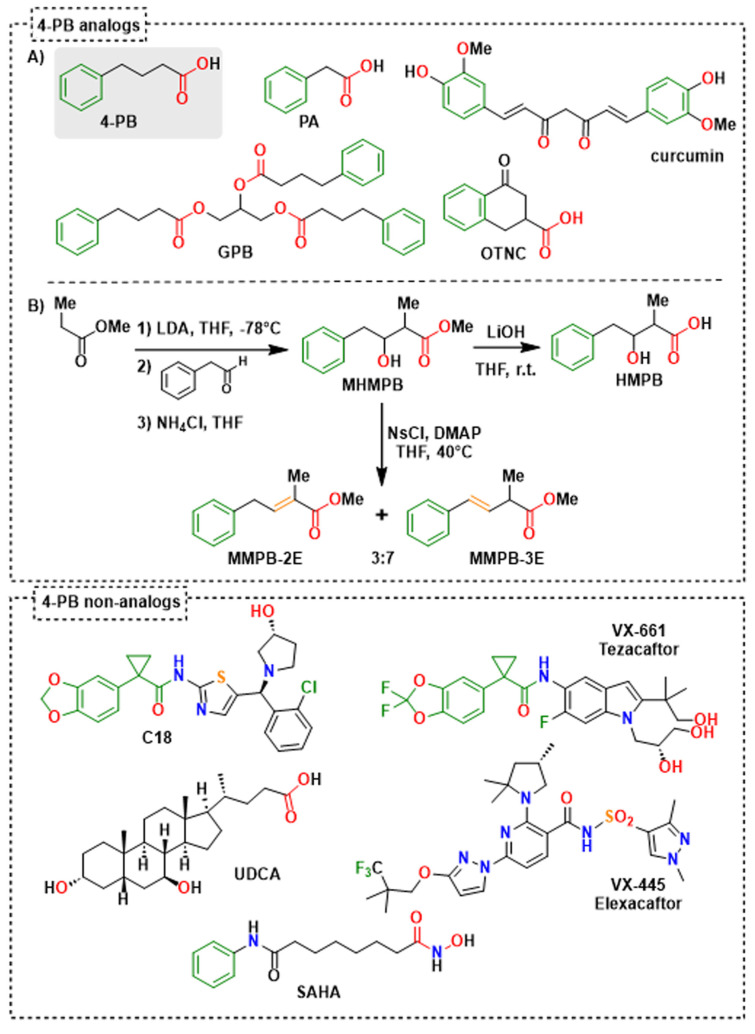
Structure of evaluated 4-PB analogs (**A**,**B**) and non-analogs, and synthesis routes of evaluated 4-PB metabolite analogs (**B**). For 4-PB analogs, the colors highlight similar pharmacophore elements: phenyl (green), alkyl chain (black), unsaturation (orange) and carboxylic group (red). For 4-PB non-analogs, only the partial structural analogy between C18 and VX-661 was highlighted (green). 4-PB: 4-phenylbutyrate; GPB: glycerol phenylbutyrate; HMPB: 3-hydroxy-2-methyl-4-phenylbutyrate; MHMPB: methyl 3-hydroxy-2-methyl-4-phenylbutanoic ester; MMPB-2E: methyl (E)-2-methyl-4-phenylbut-2-enoic ester; MMPB-3E: methyl (E)-2-methyl-4-phenylbut-3-enoic ester; OTNC: 4-oxo-1,2,3,4-tetrahydro-naphthalene-2-carboxylic acid; PA: phenylacetate; SAHA: suberoyl anilide hydroxamic acid; UDCA: ursodeoxycholic acid.

**Figure 6:**
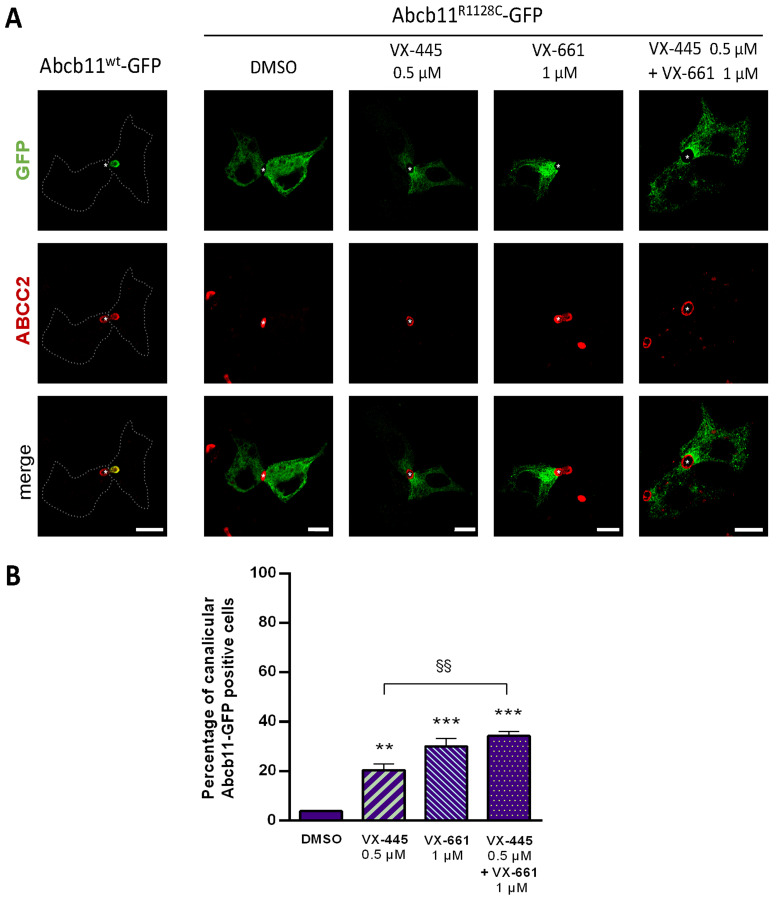
Partial rescue of the canalicular localization of Abcb11^R1128C^-GFP by elexacaftor (VX-445) and/or tezacaftor (VX-661) in hepatic polarized HepG2 cells. Cells were transiently transfected with Abcb11-GFP-encoding plasmids (wt or R1128C) and treated for 24 h at 37 °C with VX-445 and/or VX-661 or vehicle (DMSO 0.1%). (**A**) Immunolabeling of Abcb11-GFP (green) and endogenous ABCC2 (red) was visualized and analyzed using confocal microscopy. This panel is representative of at least three independent experiments per condition. Dashed lines indicate Abcb11^wt^-GFP transfected cells. Stars indicate canalicular structures. Bars: 10 µm. (**B**) Quantification of the effect of VX-445 and/or VX-661 on the canalicular expression of Abcb11^R1128C^-GFP. Among Abcb11-GFP positive cells forming canaliculi, the percentage of cells with Abcb11-GFP enrichment at the canalicular membrane was determined and expressed as means of at least three independent experiments per condition. The dashed line indicates the percentage of canalicular Abcb11-GFP positive cells of the wt protein. ** *p* < 0.01 and *** *p* < 0.001 vs. vehicle (DMSO); ^§§^ *p* < 0.01.

## Data Availability

The datasets used and/or analyzed during the current study are available on request from the corresponding author.

## Supplementary Materials

The following supporting information can be downloaded at: https://www.mdpi.com/article/10.3390/ijms26115232/s1.

## Abbreviations

The following abbreviations are used in this manuscript:

BA	Bile acid
BSEP	Bile salt export pump
CF	Cystic fibrosis
ER	Endoplasmic reticulum
GPB	Glycerol phenylbutyrate
HMPB	3-Hydroxy-2-methyl-4-phenylbutyrate
[^3^H]-TC	^3^H-Taurocholate
MHMPB	Methyl 3-hydroxy-2-methyl-4-phenyl butyric ester
MMPB-2E	Methyl (*E*)-2-methyl-4-phenylbut-2-enoic ester
MMPB-3E	Methyl (*E*)-2-methyl-4-phenylbut-3-enoic ester
OTNC	4-Oxo-1,2,3,4-tetrahydro-naphthalene-2-carboxylate
PA	Phenylacetate
4-PB	4-phenylbutyrate
PFIC2	Progressive Familial Intrahepatic Cholestasis Type 2
SAHA	Suberoylanilide hydroxamic acid
UDCA	Ursodeoxycholic acid
wt	Wild type
