# Supplementary material for: Correction of a Traffic-Defective Missense ABCB11 Variant Responsible for Progressive Familial Intrahepatic Cholestasis Type 2
Source: Int J Mol Sci. 2025 May 29;26(11):5232. doi: 10.3390/ijms26115232 (PMC12154090; doi:10.3390/ijms26115232)
Supplement: Supplementary file 1 [file ijms-26-05232-s001.zip › ijms-3626018-supplementary.pdf]

Supplementary Data

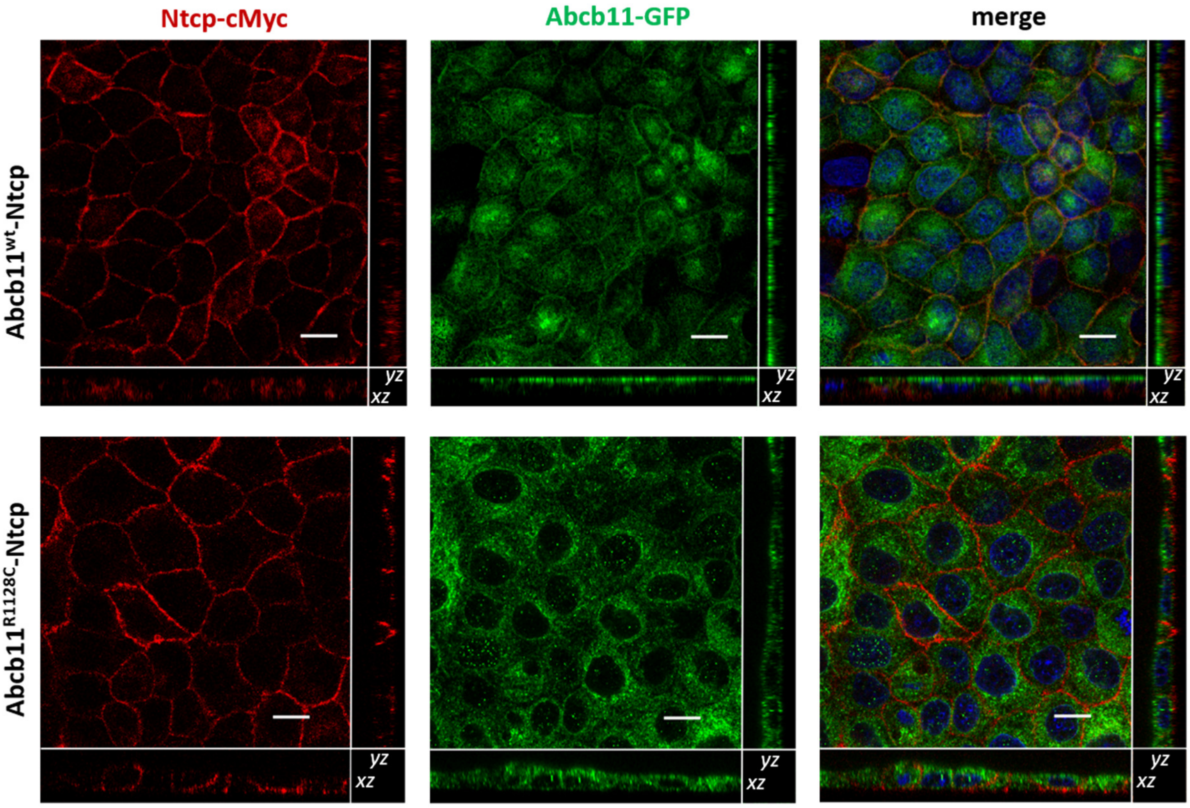

Figure S1: The Abcb11^{wt} protein is localized at the apical pole, Abcb11^{R1128C} in the cytoplasm and Ntcp on the basolateral membrane of MDCK cells. MDCK clones stably expressing Abcb11-GFP (wt or R1128C) and Ntcp-cMyc were cultured onto glass coverslips, fixed and permeabilized. Immunolabeling of Abcb11-GFP (green) and Ntcp-cMyc (red) using anti-GFP and anti-cMyc antibodies, respectively, was analyzed by confocal microscopy. Nuclei were labeled with diamidino-4',6-phénylindol-2 Dichlorhydrate (DAPI, blue). The bottom, center and right panels show x-z, x-y and y-z plan images, respectively. Bars: 10 µm.
